# Effect of Acupuncture vs Sham Acupuncture on Patients With Poststroke Motor Aphasia: A Randomized Clinical Trial

**DOI:** 10.1001/jamanetworkopen.2023.52580

**Published:** 2024-01-22

**Authors:** Boxuan Li, Shizhe Deng, Bifang Zhuo, Bomo Sang, Junjie Chen, Menglong Zhang, Guang Tian, Lili Zhang, Yuzheng Du, Peng Zheng, Gonglei Yue, Zhihong Meng

**Affiliations:** 1 National Clinical Research Center for Chinese Medicine Acupuncture and Moxibustion, Tianjin, China; 2 First Teaching Hospital of Tianjin University of Traditional Chinese Medicine, Tianjin, China; 3 Tianjin University of Traditional Chinese Medicine, Tianjin, China; 4 Air Force Medical Center of People’s Liberation Army, Beijing, China; 5 Changchun University of Chinese Medicine, Changchun, China; 6 Qilu Hospital of Shandong University, Shandong, China

## Abstract

**Importance:**

Motor aphasia is common among patients with stroke. Acupuncture is recommended as an alternative therapy for poststroke aphasia, but its efficacy remains uncertain.

**Objective:**

To investigate the effects of acupuncture on language function, neurological function, and quality of life in patients with poststroke motor aphasia.

**Design, Setting, and Participants:**

This multicenter, sham-controlled, randomized clinical trial was conducted in 3 tertiary hospitals in China from October 21, 2019, to November 13, 2021. Adult patients with poststroke motor aphasia were enrolled. Data analysis was performed from February to April 2023.

**Interventions:**

Eligible participants were randomly allocated (1:1) to manual acupuncture (MA) or sham acupuncture (SA) groups. Both groups underwent language training and conventional treatments.

**Main Outcomes and Measures:**

The primary outcomes were the aphasia quotient (AQ) of the Western Aphasia Battery (WAB) and scores on the Chinese Functional Communication Profile (CFCP) at 6 weeks. Secondary outcomes included WAB subitems, Boston Diagnostic Aphasia Examination, National Institutes of Health Stroke Scale, Stroke-Specific Quality of Life Scale, Stroke and Aphasia Quality of Life Scale–39, and Health Scale of Traditional Chinese Medicine scores at 6 weeks and 6 months after onset. All statistical analyses were performed according to the intention-to-treat principle.

**Results:**

Among 252 randomized patients (198 men [78.6%]; mean [SD] age, 60.7 [7.5] years), 231 were included in the modified intention-to-treat analysis (115 in the MA group and 116 in the SA group). Compared with the SA group, the MA group had significant increases in AQ (difference, 7.99 points; 95% CI, 3.42-12.55 points; *P* = .001) and CFCP (difference, 23.51 points; 95% CI, 11.10-35.93 points; *P* < .001) scores at week 6 and showed significant improvements in AQ (difference, 10.34; 95% CI, 5.75-14.93; *P* < .001) and CFCP (difference, 27.43; 95% CI, 14.75-40.10; *P* < .001) scores at the end of follow-up.

**Conclusions and Relevance:**

In this randomized clinical trial, patients with poststroke motor aphasia who received 6 weeks of MA compared with those who received SA demonstrated statistically significant improvements in language function, quality of life, and neurological impairment from week 6 of treatment to the end of follow-up at 6 months after onset.

**Trial Registration:**

Chinese Clinical Trial Registry: ChiCTR1900026740

## Key Points

**Question:**

What is the efficacy of acupuncture combined with language training in the treatment of motor aphasia in patients with stroke?

**Findings:**

In this randomized clinical trial involving 252 patients in China with poststroke motor aphasia, those who received 6 weeks of acupuncture treatment with up to 6 months of follow-up showed significant improvements in language function, quality of life, and neurological impairment compared with those who received sham acupuncture.

**Meaning:**

These findings suggest that acupuncture can greatly improve language function in patients with poststroke motor aphasia.

## Teaser

This randomized clinical trial investigates the effects of acupuncture on language function, neurological function, and quality of life in patients with poststroke motor aphasia.

## Introduction

Poststroke motor aphasia refers to acquired language function impairment following cerebral stroke and is characterized by chronic nonfluent speech.^1^ Among stroke survivors, approximately one-third experience aphasia during the acute stage, and 61% remain affected at 1 year after onset.^2,3,4^ Speech dysfunction in individuals with poststroke motor aphasia disrupts their communication ability and quality of life (QoL).^5,6^ In addition, aphasia is associated with more severe stroke conditions and a higher mortality rate.^7^ Given the damaging impact of aphasia on stroke prognosis and daily lives, comprehensive management of aphasia is needed. However, few management strategies have been developed to address poststroke motor aphasia. Behavioral therapy, such as language training, is recommended as a beneficial therapy,^8,9^ but patient recovery may be inhibited by physical capacity.^10^ Although pharmacotherapy for patients with aphasia is promising,^11^ its efficacy requires further investigation.^12^ As mentioned previously, patients with poststroke aphasia require additional approaches to maximize recovery.

A battery of feasible alternative approaches for poststroke aphasia has been explored,^12,13,14^ of which acupuncture is popularly applied worldwide because of its efficacy and limited adverse effects.^15^ In China, acupuncture is recommended as a complementary and alternative therapy for poststroke aphasia.^16^ Clinical studies have demonstrated the benign effects of acupuncture on speech function in patients with poststroke motor aphasia,^17^ and systematic reviews have shown the benefits of acupuncture on functional communication ability.^18,19^ Neuroimaging studies revealed strengthened connectivity within cortical-subcortical functional networks^20^ and intensified brain activation in language-related regions in patients with poststroke aphasia after receiving acupuncture,^21^ indicating the benefits of brain functional reorganization after acupuncture.

The combination of acupuncture and language training exhibited superior effects compared with acupuncture alone, but it needs further validation.^22^ In addition, a randomized, blind-controlled trial with a large sample size is required to further elucidate the clinical effects of acupuncture on poststroke motor aphasia. Hence, this study aims to investigate the effects of acupuncture on language function, QoL, and neurological impairment in patients with poststroke motor aphasia.

## Methods

### Study Design

This was a multicenter, single-blind, randomized clinical trial with 6 weeks of treatment and follow-up for up to 6 months after onset in patients with poststroke motor aphasia. Eligible participants were randomized 1:1 to receive manual acupuncture (MA) or sham acupuncture (SA). The study protocol was approved by the ethics committee of the First Teaching Hospital of Tianjin University of Traditional Chinese Medicine before participant enrollment (Supplement 1).^23^ We recruited outpatients from 3 tertiary hospitals in China, the First Teaching Hospital of Tianjin University of Traditional Chinese Medicine, the Changchun University of Chinese Medicine, and the Qilu Hospital of Shandong University, from October 21, 2019, to November 13, 2021. Written informed consent was obtained from all participants before randomization. This study follows the Consolidated Standards of Reporting Trials (CONSORT) reporting guidelines.

### Participants

Participants aged 45 to 75 years who received a diagnosis of aphasia after their first ischemic stroke (*International Statistical Classification of Diseases and Related Health Problems, Tenth Revision *code I63.902) were screened. Eligible patients were defined as those with an aphasia severity of 0 to 3 according to the Boston Diagnostic Aphasia Examination (BDAE) grading (grades range from 0 to 5, with higher grades indicating less serious language deficits) and an aphasia duration ranging from 15 to 90 days. The exclusion criteria were patients with a diagnosis of aphasia not caused by stroke, patients with aphasia before stroke onset, patients who could not complete the trial because of severe disease (severe heart disease, kidney function impairment, liver function insufficiency, dementia, or mental illness with diagnosis), patients with audiovisual impairments, and pregnant and lactating women. Dropout criteria were patients with poor compliance (those who complete <6 sessions), those who withdrew voluntarily, or those with severe adverse reactions or stroke recurrence (eTable 1 in Supplement 2).

### Randomization, Blinding, and Concealing

Participants were randomized 1:1 to the MA or SA group using the district-group randomization method. A central randomization system managed by a third-party mathematician outside the study was used to generate and conceal the allocation sequence. Nonacupuncture and nonmeridian points with shallow needle insertion were used to perform single-blinded acupuncture interventions.

### Intervention

Both MA and SA were performed for 30 sessions over 6 consecutive weeks (5 sessions per week, 30 minutes per session), combined with language training^24^ and conventional treatment.^25^ Disposable sterile needles (0.25 mm × 40 mm and 0.25 mm × 75 mm; Hwato) were used for the acupuncture intervention. MA was performed following the standard *Xing-Nao Kai-Qiao* acupuncture protocol,^26^ with a settled needling angle, depth, manipulation direction and frequency, and retention time (eTable 2 and eTable 3 in Supplement 2). Patients in the MA group received acupuncture at 8 fixed acupoints (eFigure 1 in Supplement 2): PC6 (bilateral), GV26, SP6 (bilateral), HT1 (affected side), LU5 (affected side), BL40 (affected side), CV23, and beside CV23 (bilateral). The *De Qi* sensation was induced during acupuncture stimulation. For SA, 8 sham acupoints, including nonacupoint and nonmeridian locations, were selected in a lateral opening of 1 cun (1 cun is approximately 25 mm and is defined as the width of the interphalangeal joint of the patient’s thumb) in the horizontal direction^27^ (eTable 2 in Supplement 2). Acupuncture stimulation in SA induced no *De Qi* sensation. After needle penetration, there was a 30-minute retention of the needle in both the MA and SA groups. Language training was performed in both the MA and SA groups for 30 sessions over 6 consecutive weeks (5 sessions per week, 60 minutes per session).

### Assessments

Participants’ demographic information was recorded at baseline, and pharmacologic interventions and disease-related information were recorded during the trial. The time point of the outcome assessment is illustrated in eFigure 2 in Supplement 2.

### Primary Outcome

The primary outcomes were the aphasia quotient (AQ) of the Western Aphasia Battery (WAB) and the Chinese Functional Communication Profile (CFCP) score at week 6. The AQ is a sensitive, valid, and reliable measure of aphasia performance.^28^ A lower AQ score (range, 0-100) indicates more severe impairment of language function. The CFCP measures the functional communication ability in Mandarin, with a higher score (range, 0-250) indicating a better ability.^29^

### Secondary Outcomes

The secondary outcomes included the WAB subitem scores to assess aphasia severity in aspects of spontaneous speech, auditory verbal comprehension, repetition, and naming; BDAE grade to evaluate the status of language competence^30^; National Institutes of Health Stroke Scale (NIHSS) to evaluate the severity of neurological deficits (score range, 0-42, with higher scores indicating more serious neurological impairment)^31^; Health Scale of Traditional Chinese Medicine (HSTCM) to reveal the comprehensive health condition according to the Chinese medicine theory system (score range, 0-130, with higher scores indicating worse health conditions)^32^; and Stroke-Specific Quality of Life Scale (SS-QOL) (score range, 49-245, with higher scores indicating better quality of life) and Stroke and Aphasia Quality of Life Scale–39 (SAQOL-39) (score range, 0-195, with higher scores indicating better quality of life) to assess the QoL of patients with poststroke aphasia.^33,34^ The assessments were conducted at 2, 4, 6, and 12 weeks and at 6 months after onset.

### Sample Size

The sample size calculation was described in detail in the protocol report and was determined according to the difference in AQ in the pilot study.^35^ The mean (SD) difference in the AQ score between the acupuncture and placebo groups after treatment was 10.9 (22.9). A superiority test was set to 80% with 2-sided α = .05 and β = 0.2. Considering a dropout rate of 20%, 252 participants were deemed appropriate for the trial (126 per group).

### Statistical Analysis

Data analysis was performed from February to April 2023. The baseline characteristics and outcomes were analyzed according to the intention-to-treat principle. Missing data were replaced using the multiple imputation method under the missing at random assumption.^36^ Statistical analysis was conducted on the basis of 5 imputed data sets, and the mean differences were combined to obtain a pooled effect with an associated 95% CI. Measurement data were described as mean (SD) or median (IQR), and a *t* test or nonparametric Mann-Whitney *U* test was used for analysis. Categorical data were summarized as numbers (percentages), and the χ^2^ test, Fisher exact probability method, and rank-sum sum test were used for analysis.

For the repeated measures outcomes, the dependent variable was the mean change from baseline, and the independent variables were treatment, time (visit), and treatment multiplied by time interaction. The variability between the 2 groups was tested using the Mauchly test of sphericity. If significant, the *F* test was applied; otherwise, multivariate analysis of variance was applied to determine group differences. The treatment-by-visit interaction term was determined using repeated-measures analyses of variance. If significant, the between-group differences were assessed at each time point. Otherwise, the main effects of the treatment were tested. For the comparison of each visit, Bonferroni correction was used to adjust the *P* value according to the number of tests. Subgroup analysis was conducted to test the moderating effect of baseline aphasia duration (15-30 days vs 31-90 days) on the intervention. Terms of baseline aphasia duration and the intervention group were included into the general linear model. Analyses were performed using Stata statistical software version 17.0 (Stata Corp), SPSS statistical software version 26.0 (IBM, Inc), and R statistical software version 4.1.1 (R Project for Statistical Computing). Two-sided *P* < .05 was considered statistically significant.

## Results

### Patients and Characteristics

**Figure 1.**
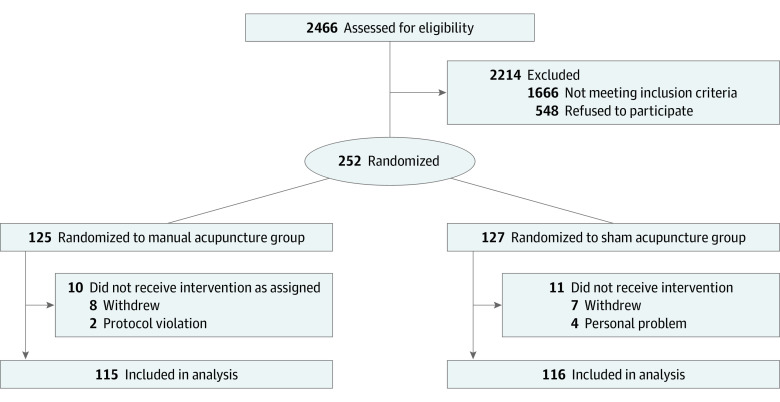
Study Flow Diagram

Initially, 2466 patients with poststroke aphasia were evaluated from October 21, 2019, to November 13, 2021; 252 eligible participants (198 men [78.6%]; mean [SD] age, 60.7 [7.5] years) were enrolled and randomized, and 231 participants (183 men [79.2%]; mean [SD] age, 60.4 [7.4] years) were included in the baseline characteristics and outcome analysis (Figure 1). Finally, 224 participants completed the follow-up assessment, and 28 participants (11.1%) dropped out (MA group, 14 [11.2%]; SA group, 14 [11.0%]). There was no significant difference in compliance between the 2 groups (eTable 4 in Supplement 2). Table 1 lists the baseline demographic and clinical characteristics of the included participants in the 2 groups, which were well balanced except for the naming score of the WAB (difference, −5.72 points; 95% CI, −11.30 to −0.13 points; *P* = .045).

**Table 1.** Baseline Characteristics of the Intention-to-Treat Population

Characteristic	Participants, No. (%) (N = 231)	
	Manual acupuncture (n = 115)	Sham acupuncture (n = 116)
Age, mean (SD), y	61.3 (7.1)	59.6 (7.7)
Sex		
Male	87 (75.7)	96 (82.8)
Female	28 (24.3)	20 (17.2)
Body mass index, mean (SD)^a^	24.60 (2.30)	24.79 (2.50)
Educational level		
Less than primary school	1 (1.0)	0
Primary school	19 (15.0)	23 (19.8)
Junior high school	37 (38.0)	41 (35.3)
Senior high school	34 (27.0)	28 (24.1)
Bachelor degree or more	24 (19.0)	24 (20.7)
Western Aphasia Battery, mean (SD), score^b^		
Aphasia quotient^c^	40.07 (13.51)	43.28 (14.75)
Spontaneous speech	5.86 (3.28)	6.25 (3.38)
Auditory verbal comprehension	128.00 (36.70)	135.24 (36.45)
Repetition	40.54 (22.35)	42.90 (22.00)
Naming^d^	37.34 (21.18)	43.05 (21.87)
Chinese Functional Communication Profile score, mean (SD), score^e^	94.91 (44.88)	101.04 (45.39)
Boston Diagnostic Aphasia Examination grade^f^		
0	11 (9.6)	10 (8.6)
1	52 (45.2)	44 (37.9)
2	35 (30.4)	43 (37.1)
3	17 (14.8)	19 (16.4)
4	0	0
5	0	0
National Institutes of Health Stroke Scale, mean (SD)^g^	9.06 (3.07)	8.79 (3.38)
Stroke-Specific Quality of Life Scale, mean (SD), score^h^	115.24 (25.88)	116.70 (29.85)
Stroke and Aphasia Quality of Life Scale–39, mean (SD)^i^		
Composite score	81.43 (22.99)	84.60 (26.42)
Mean score	2.10 (0.60)	2.17 (0.68)
Physical score	1.86 (0.90)	1.93 (0.96)
Communication score	2.00 (0.57	2.10 (0.62)
Psychological score	2.41 (0.56)	2.52 (0.61)
Health Scale of Traditional Chinese Medicine, mean (SD), score^j^	78.62 (22.14)	76.35 (19.37)

^a^ Body mass index is calculated as weight in kilograms divided by height in meters squared.

^b^ Higher scores indicate better performance.

^c^ Scores range from 0 to 100, with higher scores indicating better language function.

^d^ Statistically significant differences between the groups were detected at baseline (*P* = .045).

^e^ Scores range from 0 to 250, with higher scores indicating better communication abilities.

^f^ Grades range from 0 to 5, with higher grades indicating less serious language deficits.

^g^ Scores range from 0 to 42, with higher scores indicating more serious neurological impairment.

^h^ Scores range from 49 to 245, with higher scores indicating better quality of life.

^i^ Scores range from 0 to 195, with higher scores indicating better quality of life.

^j^ Scores range from 0 to 130, with higher scores indicating worse health conditions.

### Primary Outcome

A total of 115 of 125 participants (92.0%) were assigned to the MA group, and 116 of 127 participants (91.3%) were assigned to the SA group. The mean (SD) AQ score of the WAB at week 6 was 69.66 (17.32) in the MA group (mean [SD] improvement between baseline and week 6, 29.60 [14.07] points) and 61.68 (17.88) in the SA group. Compared with the SA group, the MA group had a significant 7.99-point increase (95% CI, 3.42 to 12.55 points; *P* < .001) in the AQ score. The mean (SD) CFCP scores at week 6 were 167.60 (45.08) in the MA group (mean [SD] improvement between baseline and week 6, 72.68 [39.56]) and 144.08 (50.52) in the SA group. Compared with the SA group, the MA group had a significant 23.51-point increase (95% CI, 11.10 to 35.93 points; *P* < .001). The MA group also showed significant improvements in AQ (difference, 10.34; 95% CI, 5.75-14.93; *P* < .001) and CFCP (difference, 27.43; 95% CI, 14.75-40.10; *P* < .001) scores at the end of follow-up at 6 months (Table 2).

**Table 2.** Primary and Secondary Outcomes

Outcome assessments	Score, mean (SD)		Difference (95% CI)	*P* value
	Manual acupuncture (n = 115)	Sham acupuncture (n = 116)		
Primary outcome at 6 wk				
Aphasia quotient	69.66 (17.32)	61.68 (17.88)	7.99 (3.42 to 12.55)	.001
Chinese Functional Communication Profile score	167.60 (45.08)	144.08 (50.52)	23.51 (11.10 to 35.93)	<.001
Secondary outcomes				
Western Aphasia Battery				
Aphasia quotient 6 mo after onset	74.69 (17.19)	64.35 (18.19)	10.34 (5.75 to 14.93)	<.001
Spontaneous speech				
6 wk	12.48 (3.40)	10.70 (3.97)	1.78 (0.83 to 2.74)	<.001
6 mo	13.58 (3.77)	11.48 (4.40)	2.10 (1.04 to 3.16)	<.001
Auditory verbal comprehension				
6 wk	172.22 (29.88)	158.00 (35.37)	14.22 (5.73 to 22.71)	.001
6 mo	178.38 (26.96)	161.48 (33.51)	16.90 (9.01 to 24.79)	<.001
Repetition				
6 wk	68.41 (24.31)	59.31 (24.19)	9.10 (2.81 to 15.38)	.005
6 mo	72.93 (24.79)	61.38 (23.76)	11.56 (5.26 to 17.85)	<.001
Naming				
6 wk	69.73 (22.42)	62.67 (23.05)	7.06 (1.16 to 12.95)	.02
6 mo	75.42 (20.53)	66.00 (22.08)	9.41 (3.89 to 14.94)	.001
Chinese Functional Communication Profile score 6 mo after onset	180.10 (45.67)	152.67 (51.86)	27.43 (14.75 to 40.10)	<.001
Boston Diagnostic Aphasia Examination grade, participants, No. (%)				
6 wk				
Grade 0	0	2 (1.7)	NA	<.001
Grade 1	9 (7.8)	11 (9.5)	NA	
Grade 2	23 (20)	38 (32.8)	NA	
Grade 3	37 (32.2)	49 (42.2)	NA	
Grade 4	43 (37.4)	15 (12.9)	NA	
Grade 5	3 (2.6)	1 (0.9)	NA	
6 mo				
Grade 0	0	2 (1.7)	NA	<.001
Grade 1	6 (5.2)	9 (7.8)	NA	
Grade 2	16 (13.9)	33 (28.4)	NA	
Grade 3	34 (29.6)	46 (39.7)	NA	
Grade 4	47 (40.9)	23 (19.8)	NA	
Grade 5	12 (10.4)	3 (2.6)	NA	
National Institutes of Health Stroke Scale				
6 wk	4.63 (3.91)	6.85 (8.55)	−2.22 (−3.95 to −0.49)	.01
6 mo	4.02 (2.12)	4.69 (2.55)	−0.66 (−1.27 to −0.05)	.03
Stroke-Specific Quality of Life Scale				
6 wk	160.17 (32.48)	148.62 (32.97)	11.55 (3.06 to 20.04)	.008
6 mo	174.60 (34.26)	162.52 (35.36)	12.08 (3.06 to 21.11)	.009
Stroke and Aphasia Quality of Life Scale–39				
Composite score				
6 wk	119.45 (29.33)	113.17 (31.24)	6.28 (−1.57 to 14.14)	.12
6 mo	133.87 (31.85)	123.73 (32.24)	10.14 (1.84 to 18.45)	.02
Mean score				
6 wk	3.05 (0.75)	2.90 (0.80)	0.15 (−0.05 to 0.35)	.14
6 mo	3.43 (0.82)	3.17 (0.83)	0.26 (0.05 to 0.47)	.02
Physical score				
6 wk	2.52 (1.53)	2.31 (1.42)	0.21 (−0.17 to 0.59)	.28
6 mo	3.27 (1.05)	3.04 (1.01)	0.23 (−0.04 to 0.49)	.10
Communication score				
6 wk	3.10 (0.73)	2.88 (0.77)	0.22 (0.03 to 0.42)	.03
6 mo	3.37 (0.85)	3.12 (0.95)	0.25 (0.02 to 0.48)	.04
Psychological score				
6 wk	3.34 (0.67)	3.14 (0.76)	0.19 (0.01 to 0.38)	.04
6 mo	3.68 (0.71)	3.37 (0.79)	0.31 (0.11 to 0.50)	.002
Health Scale of Traditional Chinese Medicine score				
6 wk	59.07 (19.12)	62.69 (17.62)	−3.63 (−8.39 to 1.14)	.14
6 mo	53.77 (19.95)	56.64 (17.56)	−2.88 (−7.75 to 1.99)	.25

Abbreviation: NA, not applicable.

**Figure 2.**
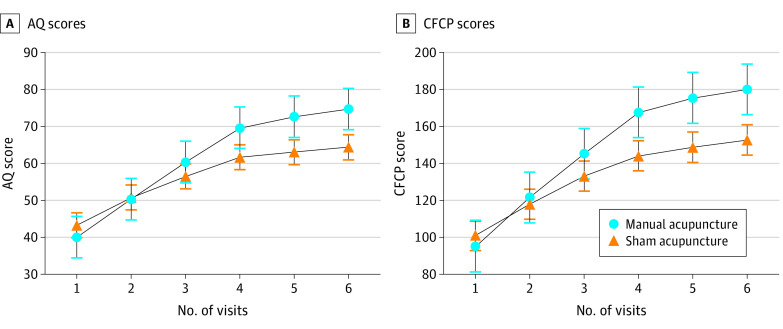
Changes in Primary Outcomes Over Time, by Group Graphs show enhancement level according to aphasia quotient (AQ) score (A) and improvement in Chinese functional communication profile (CFCP) score (B) for manual vs sham acupuncture. Error bars indicate 95% CIs.

To verify the robustness of the primary outcomes, a treatment-by-visit interaction test was performed. Significant differences were observed in the time effect, treatment effect, and treatment multiplied by time interaction in the AQ and CFCP scores (eTable 5 in Supplement 2). According to Herpich et al,^37^ neuroplasticity and cortical reorganization changes that promote functional improvement may last for 1 month. Therefore, the 30 days after onset was chosen as the time point for subgroup analysis. The results showed that the treatment effects were not moderated by baseline aphasia duration (eFigure 3 in Supplement 2). The trends in the AQ and CFCP scores are shown in Figure 2.

### Secondary Outcomes

For the subscales of the WAB, higher scores were identified in the MA group compared with the SA group with significant differences in all components, including spontaneous speech (difference, 1.78 points; 95% CI, 0.83 to 2.74 points; *P* < .001), auditory verbal comprehension (difference, 14.22 points; 95% CI, 5.73 to 22.71 points; *P* = .001), repetition (difference, 9.10 points; 95% CI, 2.81 to 15.38 points; *P* = .005), and naming (difference, 7.06 points; 95% CI, 1.16 to 12.95 points; *P* = .02), in the between-group comparison at week 6. Similar results were found in the between-group comparisons of BDAE grade, NIHSS score (difference, −2.22 points; 95% CI, −3.95 to −0.49 points; *P* = .01), SS-QOL score (difference, 11.55 points; 95% CI, 3.06 to 20.04 points; *P* = .008), and the communication score and psychological scores of the SAQOL-39, which lasted until the end of the follow-up (Table 2).

At the end of week 6, no significant differences were found in the between-group comparisons of the composite, mean, and physical scores of the SAQOL-39; however, significant differences were found at 6 months from aphasia onset for the SAQOL-39 composite score (difference, 10.14; 95% CI, 1.84 to 18.45 points; *P* = .02) and the SAQOL-39 mean score (difference, 0.26 points; 95% CI, 0.05 to 0.47 points; *P* = .02). There were no significant differences in the physical scores of SAQOL-39 and HSTCM between the groups at week 6 and 6 months after onset (Table 2).

### Adverse Events

Three adverse reactions (2.6%) occurred in the MA group, and 3 (2.6%) occurred in the SA group. However, all treatment-related adverse reactions were transient, and no serious adverse events occurred (eTable 6 and eTable 7 in Supplement 2).

## Discussion

To our knowledge, this is the first multicenter, sham-controlled, randomized clinical trial with a long-term follow-up to evaluate the efficacy of acupuncture in patients with poststroke motor aphasia. This study found that, compared with SA, 6 weeks of MA produced significant and continuous improvement in language function, QoL, and neurological impairment through 6 months after onset. The study results confirmed that poststroke motor aphasia was the dominant condition affected by the acupuncture treatment,^15^ indicating that acupuncture might serve as an adjunctive treatment for patients with poststroke motor aphasia. In addition, the clinical effects and safety results provide evidence for policymakers, clinicians, and patients regarding the management of poststroke aphasia with acupuncture.

In this study, the AQ of the WAB was chosen as the primary outcome because it interprets the integrative deficits and severity of motor aphasia.^38^ The 7.99-point between-group difference in the AQ achieved clinical meaningfulness, defined by Gilmore et al^39^ as 5.05 points, in aphasia rehabilitation. For within-group improvements, other studies on poststroke motor aphasia treatment using language training with different adjunctive therapies reported increases in the AQ ranging from 21.38 to 33.93.^35,40,41^ In this study, the 29.60-point increase in the AQ in the MA group confirmed the benefits of acupuncture in improving aphasia severity. For the CFCP, previous studies reported a 24.01-point difference in between-group comparisons,^17^ with a threshold of 26 points having minimal clinical significance and a 50-point improvement having significant clinical importance.^42^ Our results demonstrated a between-group difference of 23.51 points and a 72.68-point enhancement in the MA group after treatment, indicating the benefits of acupuncture in improving functional communication ability.

During follow-up, consistent with previous studies,^43,44^ there were continuous increases in the AQ and CFCP scores. A 15- to 24-session acupuncture treatment has been reported to be beneficial for language function deficits.^17,45^ Given the limited evidence of the specific time frame of rehabilitation for language function,^46^ some studies have reported language function evaluations ranging from 12 weeks to 6 months after intervention.^47,48^ Our results showed that the language function improvement effects of acupuncture combined with language training lasted for 6 months after onset, providing evidence for long-term assessment.

We considered possible reasons for the effect of acupuncture on improving language deficits. First, as previously reported,^15^ poststroke motor aphasia is one of the dominant conditions affected by acupuncture treatment. Second, the 30-session acupuncture treatment provided a sufficient acupuncture dose. Third, we applied treatment strictly following the standard acupuncture procedure. Recent studies have found that reorganization of the brain network is a vital mechanism underlying poststroke aphasia rehabilitation.^49^ Compared with the word generation task stimulation, patients who received acupuncture demonstrated significantly greater activation in the left middle frontal gyrus.^21^ In addition, brain functional improvements have been correlated with AQ values after acupuncture treatment,^50,51^ indicating the effects of acupuncture on brain activation. Prior research has also demonstrated the mechanistic pathways of acupuncture in the recovery from ischemic stroke, including the facilitation of neuroplasticity via neurogenesis and cell proliferation-related pathways,^52^ promotion of cerebral blood flow in the ischemic area,^53^ and reduction of cerebral ischemia and/or reperfusion damage.^54^

QoL represents the physical and psychological conditions of patients with poststroke aphasia.^55,56^ Patients with poststroke aphasia may experience psychological disorders and functional limitations.^6^ According to a study using the SS-QOL, a 4.7-point difference was significant for evaluating QoL and disease burden.^57^ In this study, the 11.55-point higher SS-QOL score in the MA group showed that acupuncture is promising for improving the QoL in patients with poststroke motor aphasia. For the SAQOL-39, higher scores on the communication and psychological subscales in the MA group indicated the benefits of acupuncture on communication function and psychological state in disease burden. However, no significant differences were found in the composite, mean, or physical-related scores of the SAQOL-39 and HSTCM at week 6. These results were consistent with those of a clinical study using multiple behavioral therapies,^58^ indicating the dominant influence of acupuncture on language function and mental health.

Regarding neurological function, after 6 weeks of treatment, there was a 2.22-point reduction in the NIHSS scores in the MA group compared with the SA group, indicating a clinical improvement in neurological impairment.^59^ The effects of acupuncture in enhancing neurological function in patients with stroke have been validated in clinical studies.^60,61^ Moreover, acupuncture is recommended by clinical practices and treatment guidelines for at least 15 poststroke symptoms worldwide.^62^ In addition, no adverse events occurred during this trial, confirming the safety of acupuncture therapy as previously reported.^63^

To facilitate the success of blinding the patients, a series of efforts were made in this trial. First, we used the SA setting coupled with *Xing-Nao Kai-Qiao* acupuncture therapy.^27^ The acupoint locations were similar in the MA and SA groups, and the needle material was the same in both groups, inducing visual blinding in the acupuncture intervention. Second, each participant received the intervention in a private compartment in the supine posture, ensuring the blinding of the patients.

### Limitations

This study had some limitations. First, some items of the WAB were designed in English, and the included patients were all Chinese speakers. Hence, bias might exist owing to the cultural gap. Second, we did not perform a blinding assessment of the participants. To assist in the success of blinding participants, we used nonacupoints with a penetrated needle.^27^ Furthermore, the acupuncture was performed in a private compartment with patients in a supine posture, ensuring blinding of the participants. In addition, to minimize anticipation bias, we introduced SA and limited the interaction between the acupuncturists and participants.

## Conclusions

In this study, patients with poststroke motor aphasia who underwent a 6-week MA treatment compared with SA demonstrated a statistically significant improvement in language function, QoL, and neurological impairment from week 6 of treatment to 6 months of follow-up after aphasia onset. Acupuncture may be considered as an adjunctive approach for patients with poststroke motor aphasia.

## Notes

Supplement 1.
Trial Protocol and Statistical Analysis Plan

Supplement 2. **eTable 1. **Inclusion Criteria, Exclusion Criteria, and Dropout Criteria **eTable 2.** Acupuncture Operation Details **eTable 3.** Acupuncture Intervention Details **eTable 4.** Results: Between-Group Comparison of Compliance of Participants **eTable 5.** Results: Treatment-by-Visit Interaction Test for the Primary Outcome^a^ **eTable 6.** Results: Adverse Event Analysis **eTable 7.** Results: Adverse Event Descriptions **eFigure 1.** Acupoint and Sham Acupoint Locations **eFigure 2.** Schedule of Enrollment, Interventions, and Assessments **eFigure 3.** Results: Subgroup Analysis

Supplement 3.
Data Sharing Statement
