# Supplementary material for: Effect of Acupuncture vs Sham Acupuncture on Patients With Poststroke Motor Aphasia: A Randomized Clinical Trial
Source: JAMA Netw Open. 2024 Jan 22;7(1):e2352580. doi: 10.1001/jamanetworkopen.2023.52580 (PMC10804271; doi:10.1001/jamanetworkopen.2023.52580)
Supplement: Supplement 1. — Trial Protocol and Statistical Analysis Plan [file jamanetwopen-e2352580-s001.pdf]

Effect of Acupuncture vs Sham Acupuncture on Patients with Poststroke Motor Aphasia: A Randomized Clinical Trial

Final study protocol

Clinical site:

1. First Teaching Hospital of Tianjin University of Traditional Chinese Medicine

2. Changchun University of Chinese Medicine

3. Qilu Hospital of Shandong University

Data management and statistical site:

Guangdong Provincial Hospital of Chinese Medicine, No.111 Dade road, Yuexiu District, Guangzhou, China

Data:

Original protocol date: December 14, 2018

Amendment date: June 27, 2019

Contents

1 Study Contact and Organization 4
1.1 Study Contacts 4
1.2 Recruiting Sites 4
2 Study Design 4
2.1 Study Overview 4
2.2 Background 5
2.3 Study Hypothesis 5
2.4 Methodology 5
2.4.1 Trial Design 5
2.4.2 Randomization and Concealment 7
2.4.3 Blinding 8
2.4.4 Sample Size 8
3 Subjects 8
3.1 Inclusion Criteria 8
3.2 Exclusion Criteria 8
3.3 Dropout criteria 9
3.4 Subject recruitment and informed consent acquirement 9
4 Outcome Measurements 9
4.1 Primary Outcome 9
4.2 Secondary Outcomes 10
4.3 Adverse events 10
5 Interventions 10

5.1 Explanation for the choice of comparators 10

5.2 Intervention description 11

5.2.1 Acupuncture and sham acupuncture 11

5.2.1.1 Manual acupuncture group 11

5.2.1.2 Sham acupuncture group 11

5.2.2 Language training 12

5.3 Strategies to improve adherence to interventions 12

6 Data management and quality control 12

7 Statistical analysis 12

8 Ethics and dissemination 13

9 Ethical Approvals of all participating hospitals 17

1 Study Contact and Organization

1.1 Study Contacts

First Teaching Hospital of Tianjin University of Traditional Chinese Medicine

Zhihong Meng

National Acupuncture and Moxibustion Clinical Medical Research Center, First Teaching Hospital of Tianjin University of Traditional Chinese Medicine, No. 88 Changling Road, Xiqing District, Tianjin 300000, China;

Phone: 18722699933

E-mail: profmengzhihong@163.com

1.2 Recruiting Sites

First Teaching Hospital of Tianjin University of Traditional Chinese Medicine

Shizhe Deng

National Acupuncture and Moxibustion Clinical Medical Research Center, First Teaching Hospital of Tianjin University of Traditional Chinese Medicine, No. 88 Changling Road, Xiqing District, Tianjin 300000, China;

Phone: 18722498627

E-mail: 18722498627@163.com

Changchun University of Chinese Medicine

Peng Zheng

Changchun University of Chinese Medicine, Boshuo Road 1035, Changchun 130117, Jilin, China

Phone: 13074317404

E-mail: zhengpeng7877@163.com

Qilu Hospital of Shandong University

Gonglei Yue

Qilu Hospital of Shandong University, No. 107, Wenhua Xilu, Jinan 250012, Shandong, China

Phone: 18560087318

E-mail: yuegonglei126@126.com

2 Study Design

2.1 Study Overview

This is a multicenter randomized sham-controlled clinical trial. We will randomly allocate 252 subjects aged 45-75 years diagnosed with poststroke aphasia into two groups in a 1:1 ratio. Patients in the experimental group will receive “Xing-Nao Kai-Qiao” acupuncture therapy, and those in the control

group will receive sham acupuncture therapy. Both groups will receive language training and conventional treatment, with a follow-up for up to six months post-onset.

2.2 Background

Poststroke motor aphasia is a common complication of ischemic stroke that leads to harmful effects in patients and is a significant economic burden on society[1]. Aphasia has been reported in approximately 21%-38% of acute ischemic stroke patients[1-3].

Acupuncture has been used in China for thousands of years as a form of Traditional Chinese Medicine therapy. It is effective in treating poststroke motor aphasia by improving functional communication and language function[4, 5]. Some studies have revealed the effects of acupuncture on spontaneous speech, repetition, naming, and communication ability in patients with poststroke motor aphasia[6, 7]. However, high-quality evidence based on well designed, sham-controlled, large-sample size randomized clinical study are needed to further prove the effects of acupuncture on poststroke motor aphasia[6, 8, 9].

Currently, multiple approaches have shown the therapeutic effects on improving poststroke aphasia. For the pharmacotherapy, studies have demonstrated the beneficial impact of medication combined with behavioral therapy[10]. However, the effectiveness of pharmacotherapy on poststroke aphasia lacks robust evidence[11]. Behavioral approaches such as speech and language therapy (SLT) or neuropsychological treatment indicate the improvement on communication ability[12]. It has been proved that language training improved oral naming accuracy for trained items in patients with aphasia, with long-term gain maintenance over time[13]. Thus, SLT may be beneficial at high intensity, at a high dose, or over a longer period[14].

The main objectives of this trial are to evaluate the efficacy and safety of acupuncture for ischemic stroke patients with poststroke motor aphasia from 15 to 90 days after the onset of aphasia.

2.3 Study Hypothesis

Acupuncture is an effective approach for treating poststroke motor aphasia, and its efficacy is superior to that of sham-acupoint acupuncture.

2.4 Methodology

2.4.1 Trial Design

This is a multicenter randomized sham-controlled clinical trial. A total of 252 participants will be randomized into manual acupuncture (MA) or sham acupuncture (SA) groups at a 1:1 ratio. Each eligible subject will undergo a 6-week treatment period, with follow-up at the end of 6 weeks of treatment and 6 months after onset. In total, six visits are scheduled for each subject at weeks 0, 2, 4, 6, and 12 and at 6 months after onset. (Table 1, Figure 1)

Table 1. Schedule of enrollment, interventions, and assessments.

	STUDY PERIOD					
	Baseline	Intervention phase			Follow-up	
TIMEPOINT	t_0	t_{2w}	t_{4w}	t_{6w}	t_{12w}	End of 6 months after the onset time
ENROLMENT:						
Eligibility screen	X					
Basic characteristic variables	X					
Informed consent	X					
Allocation	X					
INTERVENTIONS:						
Western Aphasia Battery	X	X	X	X	X	X
Boston Diagnostic Aphasia Examination	X	X	X	X	X	X
Chinese Functional Communication Profile	X	X	X	X	X	X
National Institutes of Health Stroke Scale	X	X	X	X	X	X
Stroke and Aphasia Quality of Life Scale-39	X	X	X	X	X	X
Stroke-Specific Quality of Life Scale	X	X	X	X	X	X
Health Scale of Traditional Chinese Medicine	X	X	X	X	X	X
Combined diseases and drugs		X	X	X	X	X
Adverse event		X	X	X		
Compliance		X	X	X		
Relapse					X	X

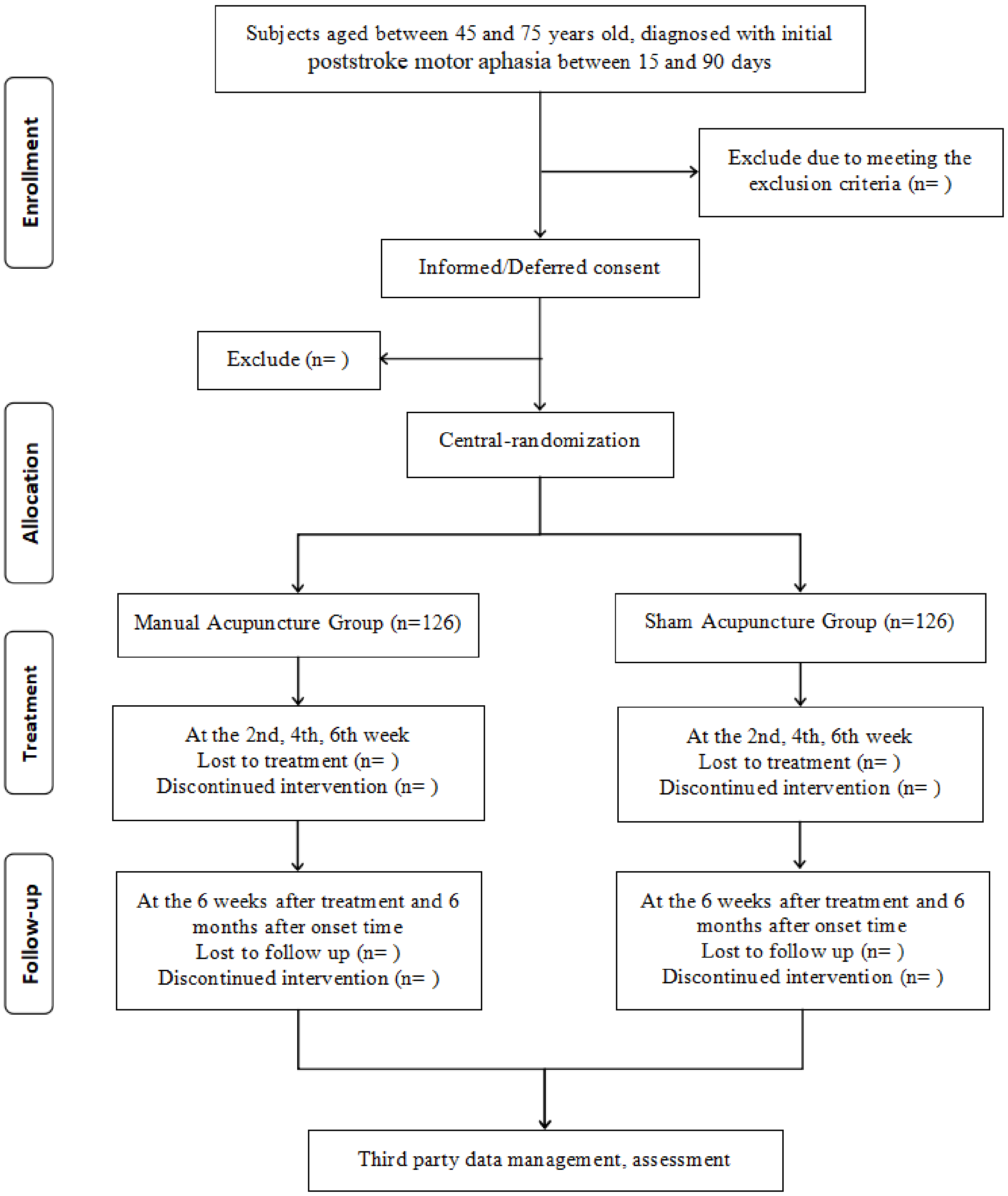

Figure 1. Study design and participation flow chart

2.4.2 Randomization and Concealment

The China Academy of Chinese Medical Sciences was commissioned to apply the "Central Randomization System for Clinical Research." The 252 participants will be randomly assigned to the experimental and control groups at a 1:1 ratio using the district-group randomization method.

The central randomization system was established to implement the allocation sequence and conceal the sequence until interventions are assigned. Throughout the study, Central will perform randomization and maintain the list, and statisticians will be continuously blinded to data management until the database is closed.

2.4.3 Blinding

Trial participants, outcome assessors, and statistical analysts will be blinded after being assigned to the intervention. Trial participants will be randomly assigned. The outcome assessor will enter the measurements into the computer, and the statistical analyst will analyze the data without access to the assignment information. The data will be reviewed in a blinded manner, entrusting third-party (Guangdong Provincial Hospital of Chinese Medicine) medical statistics professionals to conduct the statistical analysis.

2.4.4 Sample Size

Based on a previous pilot study, we expected that acupuncture combined with speech rehabilitation would be more effective than placebo combined with speech rehabilitation[6]. We assumed that the difference in the Western Aphasia Battery (WAB)-aphasia quotient (AQ) values after treatment between acupuncture and placebo was 10.9, with a standard deviation of 22.9 in both groups. According to expert clinical observations, a superiority margin of 3 was found for comparisons between acupuncture and placebo for the primary outcome. We performed a superiority test to calculate the appropriate trial sample size with 80% power, $\beta = 0.2$, and $\alpha = 0.05$. The results showed that a clinically significant difference could be detected with a sample size of at least 105 participants in each group, allowing for a predicted 20% dropout rate. We plan to enroll 252 participants in this study.

3 Subjects

Patient data will be collected throughout the clinical trials in China at three tertiary hospitals in Tianjin, Changchun, and Jinan.

3.1 Inclusion Criteria

(I) Patients diagnosed with ischemic stroke according to the International Classification of Diseases code ICD-10-I63.902[15].

(II) Patients diagnosed with aphasia for the first time after stroke.

(III) Patients diagnosed with aphasia ranging from 15 to 90 days after onset.

(IV) An aphasia severity of 0–3 that can cooperate with speech training.

(V) Consciousness and stable vital signs.

(VI) Patients aged 45-75 years and either male or female.

(VII) Patients and their families understand the research content fully, agree to participate, and signed the informed consent form.

3.2 Exclusion Criteria

(I) Patients diagnosed with aphasia not caused by stroke.

(II) Patients with aphasia occurring before stroke onset.

(III) Patients with severe heart disease and kidney and liver function insufficiency.

(IV) Patients with audiovisual dysfunction, severe cognitive impairment, or mental illness who cannot cooperate with examination and treatment.

(V) Pregnant or lactating women.

3.3 Dropout criteria

(I) Patients with poor compliance during the clinical study who are unwilling to accept the intervention or who withdraw voluntarily.

(II) Patients who experience severe adverse reactions or deterioration during the clinical study and are not eligible for further intervention.

3.4 Subject recruitment and informed consent acquirement

The study will be conducted at the First Teaching Hospital of Tianjin University of Traditional Chinese Medicine, Changchun University of Chinese Medicine, and Qilu Hospital of Shandong University. Participants diagnosed with ischemic stroke with aphasia will be recruited at three hospitals, mainly from clinical processes. Additionally, we will recruit participants through posters and flyers. Participant screening will be completed at each center by experienced researchers. Informed consent will be obtained from eligible patients, and then they will be randomized into groups.

Before the start of clinical studies, trained researchers will discuss the trial with the participants, including the research nature, research purpose, and possible benefits and dangers, thereby meeting the requirements of the *Declaration of Helsinki* and the rights and obligations of the participants. Patients will be able toparticipant in informed discussions with the researchers. The participants or their legal representatives will understand, agree to, and sign a written informed consent form indicating their willingness to participate in the trial.

4 Outcome Measurements

4.1 Primary Outcome

The main observation will be the difference between the two groups in the AQ of the WAB and the Chinese Functional Communication Profile (CFCP) score after 6 weeks of treatment.

(I) The AQ reflects the language ability of patients with aphasia according to the Research Outcome Measurement in Aphasia consensus statement recommendation[16]. A lower AQ score indicates more severe impairment of language function. Patients will be asked to answer questions about word recognition, repetition, sentence comprehension, and oral expression. Assessment time frames: at baseline; at weeks 2, 4, 6, and 12; and 6 months after onset.

(II) The CFCP represents the functional communication ability of the Mandarin language of which a higher score is related to better ability. The evaluator will ask the patients questions to assess their communication ability[17]. Assessment time frames: at baseline; at weeks 2, 4, 6, and 12; and 6 months after onset.

4.2 Secondary Outcomes

(I) Boston Diagnostic Aphasia Examination (BDAE) grading. The BDAE grade indicates the severity of the language competence status, and grades 0-5 reflect communication capacity from nonsense to full capacity. Evaluators will assess the severity of language impairment according to the patient's comprehensive condition. Assessment time frames: at baseline; at weeks 2, 4, 6, and 12; and 6 months after onset.

(II) Stroke and Aphasia Quality of Life Scale-39 (SAQOL-39)[18] score. The SAQOL-39 assesses the physical, psychological, and communication conditions in the daily lives of patients with aphasia. Participants will be asked to answer questions regarding different aspects. Assessment time frames: at baseline; at weeks 2, 4, 6, and 12; and 6 months after onset.

(III) Stroke-Specific Quality of Life Scale (SS-QOL) scores[19]. The SSQOL evaluates the activity of daily living in patients with stroke. Evaluators will ask questions to judge their quality of life. Assessment time frames: at baseline; at weeks 2, 4, 6, and 12; and 6 months after onset.

(IV) National Institutes of Health Stroke Scale (NIHSS)[20] scores. The NIHSS score reflects the neurological deficits associated with stroke. Evaluators will observe participants' physical activity and ask them to follow instructions to evaluate their condition. Assessment time frames: at baseline; at weeks 2, 4, 6, and 12; and 6 months after onset.

(V) Health Scale of Traditional Chinese Medicine (HSTCM)[21] scores. HSTCM reveals the comprehensive health condition based on the Chinese medicine theory system. Evaluators will ask questions to judge the participants' comprehensive condition. Assessment time frames: at baseline; at weeks 2, 4, 6, and 12; and 6 months after onset.

4.3 Adverse events

Adverse reactions/events will be recorded in detail, including the time of occurrence of adverse events, symptoms and signs, laboratory examination results, treatment to relieve adverse events, follow-up of adverse events, duration and severity of adverse events, and the combination of pharmaceutical therapy. For any serious adverse event occurring during the study (including events requiring hospitalization or prolonged hospitalization; events causing disability or impact on work ability, life-threatening events or death, etc.), during the clinical trial, the investigator must take appropriate treatment measures immediately and report to the ethics committee within 24 hours or no later than the second working day.

5 Interventions

5.1 Explanation for the choice of comparators

Sham acupuncture therapy is commonly used and recommended by international experts as a control in acupuncture-related randomized clinical trials. Manipulators will choose acupoints that are 1 cun away from the acupoints of the manual acupuncture group. The stimulating amount of acupuncture requires no "De Qi" sensation. Sham-acupoint acupuncture mainly involves sham acupoints that are not located on the meridians and have no therapeutic effect on aphasia.

5.2 Intervention description

To ensure consistency in interventions, qualified acupuncturists with certifications will be selected and trained on the location of acupuncture points, depth, direction of insertion, and frequency of lifting and inserting. Speech therapists will receive training related to the study prior to recruitment and speech training techniques. All acupoints will be localized according to the World Health Organization (WHO) standards. Disposable sterile acupuncture needles (0.25 mm × 40 mm, 0.25 mm × 75 mm; Hwato brand; Suzhou Medical Supplies Factory Co. LTD, Suzhou, China) will be used. Subjects in the experimental group will receive manual acupuncture, and those in the control group will receive sham acupoint acupuncture. Additionally, language training for aphasia and conventional treatment for ischemic stroke will be allowed, which are included in the *Guidelines for the Diagnosis and Treatment of Integrated Traditional Chinese and Western Medicine in Cerebral Infarction in China (2017)*[22]. The same acupuncturist in each clinical center will perform acupuncture treatment for participants in both the MA and SA groups, and the same language trainer will deliver language training for participants in both groups.

5.2.1 Acupuncture and sham acupuncture

5.2.1.1 Manual acupuncture group

"Xing-Nao Kai-Qiao" acupuncture therapy will be applied to treat poststroke motor aphasia according to traditional Chinese theory. The acupoints include bilateral PC6 (Neiguan), GV26 (Shuigou), bilateral side SP6 (Sanyinjiao), and CV23 (Lianquan), and beside CV23 (left Panglianquan and right Panglianquan), affected side HT1 (Jiquan), affected side LU5 (Chize), and affected side BL40 (Weizhong). Acupoints PC6 and GV26 are inserted at a depth of 5-10 mm, acupoint CV23 is inserted at a depth of 55 mm, and the others are inserted at a depth of 25-35 mm. After penetration, lifting-pushing and spinning-rotating manipulations will be performed to achieve the "De Qi" sensation. The acupoint location, needle method, acupuncture manipulation, and "De Qi" responses are shown in Table 2. Finally, after 30 min of retention, all needles are removed. Participants will receive treatment 5 times a week for 6 consecutive weeks.

5.2.1.2 Sham acupuncture group

Sham-acupoint acupuncture mainly involves sham acupoints that are not located on the meridians and have no therapeutic effect on aphasia. We elected the acupoints to be studied according to the WHO international standards for acupoint selection. Additionally, with the manual acupoints as a reference, the lateral opening of about 1 cun in the horizontal direction at selected nonmeridian and nonacupoint will serve as sham acupoints. The stimulating amount of sham acupuncture requires no "De Qi" sensation. The same procedure will be used as that for the MA group. The sham acupoint location, needle method, acupuncture manipulation, and "De Qi" responses in the SA group are shown in Table 2.

5.2.2 Language training

Rehabilitation language training, including listening comprehension therapy training and reading comprehension therapy training, will be used as recommended in the *Operational Specifications for Commonly Used Rehabilitation Techniques* (2012)[23]. The treatment duration will bes 60 min each at five sessions per week for six consecutive weeks. The procedure will be performed by experienced language therapists who will be professionally trained.

5.3 Strategies to improve adherence to interventions

We will improve participants' compliance by scientifically designing informed consent, strengthening humanized care, and making long-term follow-up plans. A treatment diary will be used to record the date of treatment, and the therapists will sign their names to confirm the treatment after each session. Participants will also be subsidized for transportation expenses.

6 Data management and quality control

All data will be recorded in duplicate using computer software, and different inputters will be trained. They will input the same case report and check it with each other, and the two versions will be stored in the database. Data collection should be timely, complete, and accurate. After a blind audit of the data is conducted and the established database is deemed correct, the primary researcher and statistical analyst will lock the data. Researchers will keep all study data for 5 years after the end of the study.

All information related to the study will be securely stored at the study site. Information about all participants will be held in a restricted area. All data collection, processing, and management firms will be coded and classified to keep subject information confidential. All records containing names or other personal information, such as informed consent, will be kept separate from study records. This information will be kept in a specific area.

The quality supervisor will regularly verify the test process and check the authenticity of the data to ensure the quality of the test. Meanwhile, the data will be independently managed by a third party.

The principal investigator and study physicians will design, conduct, and prepare protocols, investigator manuals, and case report forms (CRFs). In addition, they will organize steering committee meetings, manage the clinical trial office, and publish study reports. They are members of the trial management committee. The steering committee will consist of a manager from the project contractor, and each subcenter will be responsible for recruiting subjects, advancing the study's progress, and ensuring its successful completion. The study team will be responsible for subject recruitment, collection of trial data, completion of the CRF, and completion of subject follow-up according to the study protocol and investigator manual. The data manager is responsible for data entry and validation.

7 Statistical analysis

Baseline characteristics of the patients will be summarized into groups according to the study data. The chi-square test will be used to compare categorical variables, and the 2-sample *t* test or Wilcoxon rank-sum test will be used to compare continuous variables. Analysis of variance for repeated measures will be applied according to the outcome of the measurement time, or linear mixed models will be used

to analyze changes in measurements at different time points. Covariates such as age, duration of illness, and severity of aphasia will be included in the statistical model to correcting for the effects of possible imbalances. If the distribution assumption is violated, and appropriate transformation will be used.

Data analysis will be performed based on the intention-to-treat analysis (ITT) and per-protocol analysis (PP) principles. Missing outcome data will be replaced using the last observation carried forward (LOCF) or multiple imputation method. We will also assess the robustness of the results of our research by comparing the results of the two datasets mentioned above to evaluate group effects.

8 Ethics and dissemination

This study was approved by the ethics committee of the First Teaching Hospital of Tianjin University of Traditional Chinese Medicine and is registered on ChiCTR with the ID ChiCTR1900026740. Informed consent will be obtained from all included participants before randomization. The results of this trial will be published in a peer-reviewed academic journal.

Table 2. Acupuncture operation details.

	Acupoint	Acupoint location	Needle method	Acupuncture manipulation	De Qi response
Manual acupuncture group	Shuigou (GV26)	On the face, at the intersection of the upper one-third and middle one-third of the philtrum midline	Insert at a depth of 5–10 mm	Pecking technique to achieve an intense 6-9-s stimulation	Deep ache and throbbing
	Neiguan (PC6)	In the volar aspect of the forearm, at the junction of Quze (PC3) and Daling (PC7), 2 cun above the wrist crease in the space between tendon palmaris longus and vagina tendinis musculi flexoris carpi radialis	Insert at a depth of 5–10 mm	Counterclockwise at the left side and clockwise at the right side with twisting force, together with light insertion and heavy lifting, for 1 minute	Numbness sensation spreading on forearm
	Weizhong (BL40)	At the midpoint of the popliteal crease, at the midpoint between the femoral biceps tendon and the semitendinosus tendon	Insert at a depth of 25–35 mm	Light insertion and heavy lifting manipulation	Make the affected leg twitch three times
	Lianquan (CV23)	In the anterior region of the neck, superior to the superior border of the thyroid cartilage, in the depression superior to the hyoid bone on the anterior median line	Insert at a depth of 55 mm	Light insertion and heavy lifting manipulation	Numbness sensation spreading to the tongue
	Panglianquan (beside CV23)	At 0.5 cun apart from both sides of CV23	Insert at a depth of 25–35 mm	Light insertion and heavy lifting manipulation	Numbness sensation spreading to the tongue

	Jiquan (HT1)	In the axilla, in the center of the axillary fossa, over the axilary artery	Insert at a depth of 25–35 mm	Light insertion and heavy lifting manipulation	Make the affected arm twitch three times.
	Chize (LU5)	In the cubital crease, at the depression of the bicipital muscle tendon radialis at the elbow	Insert at a depth of 25–35 mm	Light insertion and heavy lifting manipulation	Make the affected hand rotate outward and twitch three times
	Sanyinjiao (SP6)	In crus inside, 3 cun above the upper border of the medial malleolus of the ankle at the posterior border of the tibia	Insert at a depth of 25–35 mm	Heavy insertion and light lifting manipulation	Make the affected leg twitch three times
Sham acupuncture group	Sham PC6	In the volar aspect of the forearm, 2 cun above the wrist crease, between the flexor carpi radialis tendon and brachioradialis tendon	Insert needle in 0.2 cun	Without needle manipulation	Deep ache or dull ache, without spreading
	Sham GV26	On the face, by the sides of GV26 1cun, above the corner of the mouth	Insert needle toward the nasal septum in 0.2 cun		
	Sham SP6	In crus inside, 3 cun above the tip of the medial malleolus, at the anterior border of the tibia, about 1.25 cun in front of SP6	Insert needle along with the medial border of the tibia in 0.2 cun		
	Sham HT1	Extend the upper arm, 1 cun below the apex of the armpit, about 1 cun forward, avoiding the armpit hair, over the abdomen of the biceps	Insert needle in 0.2 cun		
	Sham LU5	Bend the elbow 120°, at the midpoint of the line between LU5 and LI11	Insert needle in 0.2 cun		
	Sham BL40	On the belly of peroneus longus, at the inferolateral of BL40. Inferior to BL40 1cun and lateral to 1cun	Insert needle in 0.2 cun		

	Sham CV23	At the rear of CV23, and by the sides of the median line 1cun	Insert needle in 0.2 cun		
	Sham beside CV23	At the rear of beside CV23, and by the sides of the median line 2cun	Insert needle in 0.2 cun		

[a]cun: 1 cun (≈25 mm) is defined as the width of the interphalangeal joint of the patient’s thumb.

9 Ethical Approvals of all participating hospitals

This trial will be conducted at three hospitals. The ethical review was firstly submitted to the ethics committee of the principal organization, the First Teaching Hospital of Tianjin University of Traditional Chinese Medicine, and then to the ethics committees of the other participating hospitals. This trial was approved by all of the ethics committees. Ethical approvals are attached as follows.

1. First Teaching Hospital of Tianjin University of Traditional Chinese Medicine
2. Changchun University of Chinese Medicine
3. Qilu Hospital of Shandong University

Independent Ethics Committee (IEC) of The First Teaching Hospital of Tianjin University of Traditional Chinese Medicine

Approval Notice

Approval Number: TYLL2019[K]No. 015

According to ethical principle of "*Ethical Review Methods for Biomedical Study Involving Human Subjects*" (2016), "*Management Specifications for Ethical Review of Traditional Chinese Medicine Clinical Study*"(2010) issued by National Administration of Traditional Chinese Medicine, "*Guidelines for Ethical Review Work of Drug Clinical Trials*"(2003) issued by National Food and Drug Administration of the People's Republic of China, and "*Declaration of Helsinki*"(2013) issued by The World Medical Association, "*International Ethical Guidelines for Biomedical Research Involving Human Subjects*" issued by Council for International Organizations of Medical Sciences.

After review by the Ethics Committee of the First Teaching Hospital of Tianjin University of Traditional Chinese Medicine on August 27, 2019, the project "A randomized controlled trial for 'Xing-Nao Kai-Qiao' Rehabilitation Program in the Treatment of Motor Aphasia after Stroke" was approved by the ethical committee of the First Teaching Hospital of Tianjin University of Traditional Chinese Medicine, and the project investigator, Zhihong Meng, should be responsible for the clinical research project.

Sponsors and researchers are requested to conduct the clinical study in strict compliance with the Good Clinical Practice regulations and the protocol approved by this Ethics Committee (Version 1, Date: 20190627), Informed Consent Form (Version 1, Date: 20190627). Clinical trial registration (including registration in the Medical Research Registration and Filing Information System) must be completed prior to the commencement of the study. If any of the following occurs during the project, a written report should be submitted to the ethical committee: (1) any modification to the clinical protocol, informed consent, etc.; (2) replacement of the principal investigator; (3) occurrence of serious adverse events; (4) occurrence of any situation that may affect the conduct of the trial or increase the risk to the subjects; (5) violation of the protocol; (6) suspension or early termination of the clinical study.

The Ethics Committee will follow up on this project with a frequency of 12 months. Please submit a progress report by July 27, 2020. Upon completion of the project, please submit a final report.

This approval is valid from August 27, 2019 to August 27, 2022.

IEC of The First Teaching Hospital of Tianjin University of Traditional Chinese Medicine

Date: Aug 27, 2019

天津中医药大学第一附属医院医学伦理委员会

IEC of The First Affiliated Hospital of Tianjin University of Traditional Chinese Medicine

审 查 批 件

Approval Notice

伦理批件号：TYLL2019[K]字 015

根据卫生部《涉及人的生物医学研究伦理审查办法》（2016）、国家中医药管理局《中医药临床研究伦理审查管理规范》（2010）、国家食品药品监督管理局《药物临床试验伦理审查工作指导原则》（2010）、《药物临床试验质量管理规范》（2003），以及世界医学会《赫尔辛基宣言》（2013）、国际医学科学组织理事会《人体生物医学研究国际伦理指南》（2016）的伦理原则，经天津中医药大学第一附属医院医学伦理委员会 2019 年 8 月 27 日会议审查，同意由申办者天津中医药大学第一附属医院和主要研究者孟智宏共同申请的“醒脑开窍”康复方案治疗中风后运动性失语的循证研究项目开展临床研究工作。

请申办者、研究人员严格遵循 GCP 规定和本伦理委员会批准的方案（版本号：第 1 版 版本日期：20190627）、知情同意书版本号：（版本号：第 1 版 版本日期：20190627）开展临床研究。在研究开始前，须完成临床试验注册（包括医学研究登记备案信息系统登记）。该项目进行中如发生下列情况，须及时书面报告本伦理委员会：①对临床方案、知情同意书等的任何修改；②更换主要研究者；③发生严重不良事件；④出现任何可能影响试验进行或增加受试者危险的情况；⑤出现违反方案情况；⑥暂停或提前终止临床研究。

本伦理委员会将对该项目进行跟踪审查，跟踪审查频率为 12 个月，请于 2020 年 7 月 27 日前提交研究进展报告。项目完成后，请提交结题报告。

本批件有效期为 2019 年 8 月 27 日至 2022 年 8 月 27 日。

天津中医药大学第一附属医院医学伦理委员会

主任委员签字：[illegible]

日　　期：2019.8.27

联系人：贾景蕴　　联系电话：022-27986258

355

Ethical Approval

Approval Number: CCZYFYLL2019 No. 035-01

Review date	July 14, 2020
Location of review	No. 1478 Gongnong Avenue, Changchun City
Clinical research approvals	NA
Clinical Research Program	A randomized controlled trial for “Xing-Nao Kai-Qiao” Rehabilitation Program in the Treatment of Motor Aphasia after Stroke
Review of documentation	informed consent
Contract Research Organization/CRO	The Hospital of Changchun University of Chinese Medicine
Clinical Research Organization	The Hospital of Changchun University of Chinese Medicine
Principal Investigator	Peng Zheng, Yanan Zhang, Lei Liu, Jinting Wu, Tingting Liu
Review methods	■Conference review ☐Expedited review
Reviewers	Jixiang Ren, Zhuang Xiong, Haitao Li, Shulin Lian, Lijuan Yuan, Xianchen Meng
Review comments	According to “Ethical Review Methods for Biomedical Study Involving Human Subjects” issued by the National Health Commission of the People's Republic of China, “Good Clinical Practice” enacted by the National Food and Drug Administration of the People’s Republic of China in 2020, and “Declaration of Helsinki”, “International Ethical Guidelines for Biomedical Research Involving Human Subjects” issued by Council for International Organizations of Medical Sciences. After reviewing by this Ethics Committee, we agree to conduct the clinical trial of A randomized controlled trial for ‘Xing-Nao Kai-Qiao’ Rehabilitation Program in the Treatment of Motor Aphasia after Stroke at the Hospital of Changchun University of Chinese Medicine. This approval will be filed with the Institutional and Ethics Committee of the Center. If there is any disagreement about the feasibility of the program in your institution (including the qualifications and experience of the investigators, equipment and conditions, etc.), please contact the Ethics Committee timely.

	This approval is valid for 2 years from the date of issuance, and the researcher must strictly use the text of the informed consent form and the research protocol approved by the ethical committee. If the clinical studies (including statistical analyses) cannot be completed at the time of expiration of the ethical approval, please submit a request for a follow-up review one month before the expiration of the approval. Please submit the follow-up application 1 month before July 20, 2021. If the study is completed and within the validity period of the approval, a final report should be submitted to the Ethics Committee. Suspension or early termination of the clinical study should be notified to the Ethics Committee timely. Serious adverse events and unintended adverse events affecting the risk-benefit ratio of the study should be reported to the Ethics Committee. Any modification of the clinical research protocol and informed consent form, including the change of principal investigator, etc., should be submitted to the Ethics Committee for re-examination and approval before implementation. Protocol violations that affect subjects' willingness to participate in the study should be reported promptly.		
Approval validity date	July 20, 2020 to July 20, 2022	Contact	Hongwei Gao: 0431-86177876
			The Hospital of Changchun University of Chinese Medicine<br>Date : July 20, 2020

伦理审查批件

审查号：CCZYFYLL2019 审字-035-01

审查日期	2020 年 07 月 14 日		
审查地点	长春市工农大路 1478 号		
临床研究批文	NA		
临床研究项目	“醒脑开窍”康复方案治疗中风后运动性失语的循证研究		
审查文件	■知情同意书		
申办者/CRO	长春中医药大学附属医院		
临床研究单位	长春中医药大学附属医院		
主要研究者	郑鹏、张亚男、刘磊、吴今婷、刘婷婷		
伦理审查方式	■会议审查　　□快速审查		
审查人员	任吉祥、熊壮、李海涛、连树林、袁丽娟、孟宪臣		
审查意见	根据国家卫生健康委员会颁布的《涉及人的生物医学研究伦理审查办法》、中华人民共和国国家药品监督管理局 2020 年颁布实施的《药物临床试验质量管理规范》以及《赫尔辛基宣言》和国际医学科学组织委员会颁布的《人体生物医学研究国际道德指南》等伦理准则，经本伦理委员会审查，同意在长春中医药大学附属医院进行““醒脑开窍”康复方案治疗中风后运动性失语的循证研究”临床试验。本批件将在本中心机构及其伦理委员会备案。如果对方案在本机构的可行性（包括研究者的资格与经验、设备与条件等）有不同意见，请及时与本伦理委员会联系。<br>本审查自签发日期有效期 2 年，研究负责人必须严格使用经审查同意的知情同意书文本和研究方案。如伦理审查批件失效时不能完成所有的临床研究（包括统计分析），请在本批件失效前一个月，递交跟踪审查申请。请于 2021 年 07 月 20 日前 1 个月提交跟踪审查；如研究结束并在审查有效期内，请向本伦理委员会提交研究结题报告；暂停/提前终止临床研究，请及时通知伦理委员会；如发生严重不良事件以及影响研究风险受益比的非预期不良事件，应及时报告本伦理委员会；临床研究方案、知情同意书的任何修改，包括主要研究者的更换等，需递交研究方案修改申请表，经伦理委员会重新审查，获得批准后执行；发现影响受试者参加研究意愿的违反方案情况应及时报告。		
批件有效期	2020 年 07 月 20 日～2022 年 07 月 20 日	联系电话	高宏伟：0431-86177876

主任委员签字：

伦理委员会盖章

日期：2020 年 07 月 20 日

359

Independent Ethics Committee (IEC) of Qilu Hospital of Shandong University

Approval of Clinical Program

Approval Number: No. 2019 (142)

Project Title	A randomized controlled trial for “Xing-Nao Kai-Qiao” Rehabilitation Program in the Treatment of Motor Aphasia after Stroke						
Funding	National Key Research and Development Program (2018YFC1706001)						
Applicant Section	Acupuncture and Tuina Section		Applicator	Gonglei Yue			
List of Ethics Committees	gender	specialized field	professional title	agree	Agree after modification	disagree	sign
Chairperson Hui Tian	male	surgery	chief physician	√			Hui Tian
Vice-chairman Jie Yang	male	pediatrics	chief physician	√			Jie Yang
Vice-chairman Laidong Dong	female	administrative management	Associate Chief Nurse	√			Laidong Dong
Council Jun Tian	male	surgery	chief physician	√			Jun Tian
Council Min Zhu	male	surgery	chief physician	√			Min Zhu
Council Li Ma	female	administrative management	associate editor	√			Li Ma
Council Yongjuan Xu	female	jurisprudence	solicitors	√			Yongjuan Xu
Council Anchang Liu	male	pharmacy	Chief Pharmacist	√			Anchang Liu
Council Xiquan Zhang	male	surgery	chief physician	√			Xiquan Zhang

Council<br>Xiaorong Luan	female	Nursing	Chief Nurse	√			Xiaoron<br>g Luan
Council<br>Jun Peng	male	internal medicine	chief<br>physician	√			Jun Peng

Approval comments： (one) agree√

(two) Agree after modification

(three) disagree

July 4, 2019

山东大学齐鲁医院医学伦理委员会

临床项目审批件

（ 科 ）伦审第 2019（ 142 ）号

项目名称	"醒脑开窍"康复方案治疗中风后运动性失语的循证研究						
目的	国家重点研发计划（2018YFC1706001）						
申请科室	针灸推拿科		负责人	岳公雷			
伦理委员会名单	性别	专业	职 称	同意	修改后同意	不同意	签 名
主任委员 田 辉	男	外科学	主任医师	√			田辉
副主任委员 杨 杰	男	儿科学	主任医师	√			杨杰
副主任委员 董来东	女	行政管理	副主任护师	√			董来东
委员 田 军	男	外科学	主任医师	√			田军
委员 朱 民	男	外科学	主任医师	√			朱民
委员 马 莉	女	行政管理	副编审	√			马莉
委员 许永娟	女	法学	律 师	√			许永娟
委员 刘安昌	男	药学	主任药师	√			刘安昌
委员 张希全	男	外科学	主任医师	√			张希全
委员 栾晓嵘	女	护理学	主任护师	√			栾晓嵘
委员 彭 军	男	内科学	主任医师				

审批意见：（一）同意 √
（二）做必要修改后同意
（三）不同意

主任委员签名：田辉

2019 年 07 月 09 日

山东大学齐鲁医院医学伦理委员会

References:

1. Wade DT, Hewer RL, David RM, Enderby PM. Aphasia after stroke: natural history and associated deficits. *Journal of neurology, neurosurgery and psychiatry*. 1986;49(1):11-16. doi:10.1136/jnnp.49.1.11

2. Engelter ST, Gostynski M, Papa S, et al. Epidemiology of aphasia attributable to first ischemic stroke. *STROKE*. 2006;37(6):1379-1384. doi:10.1161/01.STR.0000221815.64093.8c

3. Pedersen PM, Stig Jørgensen H, Nakayama H, Raaschou HO, Olsen TS. Aphasia in acute stroke: incidence, determinants, and recovery. *ANN NEUROL*. 1995;38(4):659-666. doi:10.1002/ana.410380416

4. Zhang B, Han Y, Huang X, et al. Acupuncture is effective in improving functional communication in post-stroke aphasia. *WIEN KLIN WOCHENSCHR*. 2019;131(9-10):221-232. doi:10.1007/s00508-019-1478-5

5. Murray LL, Kim H. A review of select alternative treatment approaches for acquired neurogenic disorders: relaxation therapy and acupuncture. *SEMIN SPEECH LANG*. 2004;25(2):133. doi:10.1055/s-2004-825651

6. J M, LX F, YM L, YL R, JY H, YZ Q. Clinical observation on the xing nao kai qiao acupuncture plus language rehabilitation training for motor aphasia caused by cerebral infarction: a report of 30 cases. *J TRADIT CHIN MED*. 2010;51(05):428-431.

7. Zhao Q. Clinical study of xing nao kai qiao acupuncture treatment on motor aphasia after stroke. *Journal of Sichuan Traditional Chinese Medicine*. 2011;29(10):120-121.

8. Wu Q, Hu X, Wen X, Li F, Fu W. Clinical study of acupuncture treatment on motor aphasia after stroke. *TECHNOL HEALTH CARE*. 2016;24(s2):S691-S696. doi:10.3233/THC-161197

9. Pang Y, Wu LB, Liu DH. [Acupuncture therapy for apoplectic aphasia: a systematic review]. *Zhongguo Zhen Jiu*. 2010;30(7):612-6.

10. Greener J, Enderby P, Whurr R. Pharmacological treatment for aphasia following stroke.

COCHRANE DB SYST REV. 2001. doi:10.1002/14651858.CD000424

11. Greener J, Enderby P, Whurr R. Pharmacological treatment for aphasia following stroke. *COCHRANE DB SYST REV*. 2001;2001(4):CD000424. doi:10.1002/14651858.CD000424

12. Berube S, Hillis AE. Advances and innovations in aphasia treatment trials. *STROKE*. 2019;50(10):2977-2984. doi:10.1161/STROKEAHA.119.025290

13. Cotelli M, Manenti R, Ferrari C, Gobbi E, Macis A, Cappa SF. Effectiveness of language training and non-invasive brain stimulation on oral and written naming performance in primary progressive aphasia: a meta-analysis and systematic review. *Neuroscience & Biobehavioral Reviews*. 2020;108(498-525. doi:10.1016/j.neubiorev.2019.12.003

14. Brady MC, Kelly H, Godwin J, Enderby P. Speech and language therapy for aphasia following stroke. *COCHRANE DB SYST REV*. 20125):CD000425. doi:10.1002/14651858.CD000425.pub3

15. Regier DA, Kaelber CT, Roper MT, Rae DS, Sartorius N. The icd-10 clinical field trial for mental and behavioral disorders: results in canada and the united states. *AM J PSYCHIAT*. 1994;151(9):1340-50. doi:10.1176/ajp.151.9.1340

16. Wallace SJ, Worrall L, Rose T, et al. A core outcome set for aphasia treatment research: the roma consensus statement. *INT J STROKE*. 2019;14(2):180-185. doi:10.1177/1747493018806200

17. Wang X, Zhuo D. The chinese functional communication profile and its value in the evaluation of aphasia following stroke ii. Comparison with bdae. *Chinese Journal of Rehabilitation Medicine*. 1993;008(2):60-63.

18. Qiu W, Guan H, Chen Z, et al. Psychometric properties of the chinese-version stroke and aphasia quality of life scale 39-generic version (saqol-39g). *TOP STROKE REHABIL*. 2019;26(2):106-112. doi:10.1080/10749357.2018.1544842

19. Hilari K, Byng S. Measuring quality of life in people with aphasia: the stroke specific quality of life scale. *INT J LANG COMM DIS*. 2001;36 Suppl(86-91.

doi:10.3109/13682820109177864

20. Brott T, Adams HJ, Olinger CP, et al. Measurements of acute cerebral infarction: a clinical examination scale. *STROKE*. 1989;20(7):864-70. doi:10.1161/01.str.20.7.864

21. DR W, SL L, XF G, ZH W, WX L. Preliminary evaluation of the measurement characteristics of tcm health scale. *J Chin Integr Med*. 2008;7(6):682-9.

22. Gao C, Wu C, Zhao J, et al. Guidelines for the diagnosis and treatment of integrated traditional chinese and western medicine in cerebral infarction in china(2017). *J Chin Integr Med*. 2018;38(02):136-144. doi:10.7661/j.cjim.20171221.483

23. Chinese Association Of Rehabilitation Medicine. *Operational specifications for commonly used rehabilitation techniques (2012 edition)*. Beijing: China Woman Publishing House; 2012.
