# Supplementary material for: Effect of Acupuncture vs Sham Acupuncture on Patients With Poststroke Motor Aphasia: A Randomized Clinical Trial
Source: JAMA Netw Open. 2024 Jan 22;7(1):e2352580. doi: 10.1001/jamanetworkopen.2023.52580 (PMC10804271; doi:10.1001/jamanetworkopen.2023.52580)
Supplement: Supplement 2. — eTable 1. Inclusion Criteria, Exclusion Criteria, and Dropout Criteria eTable 2. Acupuncture Operation Details eTable 3. Acupuncture Intervention Details eTable 4. Results: Between-Group Comparison of Compliance of Participants eTable 5. Results: Treatment-by-Visit Interaction Test for the Primary Outcomea eTable 6. Results: Adverse Event Analysis eTable 7. Results: Adverse Event Descriptions eFigure 1. Acupoint and Sham Acupoint Locations eFigure 2. Schedule of Enrollment, Interventions, and Assessments eFigure 3. Results: Subgroup Analysis [file jamanetwopen-e2352580-s002.pdf]

Supplemental Online Content

Li B, Deng S, Zhou B, et al. Effect of acupuncture vs sham acupuncture on patients with poststroke motor aphasia: a randomized clinical trial. *JAMA Netw Open*. 2024;7(1):e2352580. doi:10.1001/jamanetworkopen.2023.52580

eTable 1. Inclusion Criteria, Exclusion Criteria, and Dropout Criteria

eTable 2. Acupuncture Operation Details

eTable 3. Acupuncture Intervention Details

eTable 4. Results: Between-Group Comparison of Compliance of Participants

eTable 5. Results: Treatment-by-Visit Interaction Test for the Primary Outcome[a]

eTable 6. Results: Adverse Event Analysis

eTable 7. Results: Adverse Event Descriptions

eFigure 1. Acupoint and Sham Acupoint Locations

© 2024 Li B et al. *JAMA Network Open.*

eFigure 2. Schedule of Enrollment, Interventions, and Assessments

eFigure 3. Results: Subgroup Analysis

This supplemental material has been provided by the authors to give readers additional information about their work.

© 2024 Li B et al. *JAMA Network Open.*

eTable 1. Inclusion Criteria, Exclusion Criteria, and Dropout Criteria

Inclusion criteria	(I) Patients diagnosed with ischemic stroke according to the International Classification of Diseases code ICD-10-I63.902.<br>(II) Patients diagnosed with aphasia for the first time after stroke.<br>(III) Patients diagnosed with aphasia ranging from 15 to 90 days after onset.<br>(IV) An aphasia severity of 0–3 that can cooperate with speech training.<br>(V) Consciousness and stable vital signs.<br>(VI) Patients aged 45-75 years and either male or female.<br>(VII) Patients and their families understand the research content fully, agree to participate, and signed the informed consent form.
Exclusion criteria	(I) Patients diagnosed with aphasia not caused by stroke.<br>(II) Patients with aphasia occurring before stroke onset.<br>(III) Patients with severe heart disease and kidney and liver function insufficiency.<br>(IV) Patients with audiovisual dysfunction, severe cognitive impairment, or mental illness who cannot cooperate with examination and treatment.<br>(V) Pregnant or lactating women.
Dropout criteria	(I) Patients with poor compliance during the clinical study who are unwilling to accept the intervention or who withdraw voluntarily.<br>(II) Patients who experience severe adverse reactions or deterioration during the clinical study and are not eligible for further intervention.

© 2024 Li B et al. *JAMA Network Open.*

eTable 2. Acupuncture Operation Details

	Acupoint	Acupoint location	Needle method	Acupuncture manipulation	De Qi response
Manual acupuncture group	Shuigou (GV26)	On the face, at the intersection of the upper one-third and middle one-third of the philtrum midline	Insert at a depth of 5–10 mm	Pecking technique to achieve an intense 6-9-s stimulation	Deep ache and throbbing.
	Neiguan (PC6)	In the volar aspect of the forearm, at the junction of Quze (PC3) and Daling (PC7), 2 cun above the wrist crease in the space between tendon palmaris longus and vagina tendinis musculi flexoris carpi radialis	Insert at a depth of 5–10 mm	Counterclockwise at the left side and clockwise at the right side with twisting force, together with light insertion and heavy lifting, for 1 minute	Numbness sensation spreading on forearm
	Weizhong (BL40)	At the midpoint of the popliteal crease, at the midpoint between the femoral biceps tendon and the semitendinosus tendon	Insert at a depth of 25–35 mm	Light insertion and heavy lifting manipulation	Make the affected leg twitch three times
	Lianquan (CV23)	In the anterior region of the neck, superior to the superior border of the thyroid cartilage, in the depression superior to the hyoid bone on the anterior median line	Insert at a depth of 55 mm	Light insertion and heavy lifting manipulation	Numbness sensation spreading to the tongue

© 2024 Li B et al. *JAMA Network Open.*

	Panglianquan(beside CV23)	At 0.5 cun apart from both sides of CV23	Insert at a depth of 25–35 mm	Light insertion and heavy lifting manipulation	Numbness sensation spreading to the tongue
	Jiquan (HT1)	In the axilla, in the center of the axillary fossa, over the axilary artery	Insert at a depth of 25–35 mm	Light insertion and heavy lifting manipulation	Make the affected arm twitch three times.
	Chize (LU5)	In the cubital crease, at the depression of the bicipital muscle tendon radialis at the elbow	Insert at a depth of 25–35 mm	Light insertion and heavy lifting manipulation	Make the affected hand rotate outward and twitch three times
	Sanyinjiao (SP6)	In crus inside, 3 cun above the upper border of the medial malleolus of the ankle at the posterior border of the tibia	Insert at a depth of 25–35 mm	Heavy insertion and light lifting manipulation	Make the affected leg twitch three times
Sham acupuncture group	Sham PC6	In the volar aspect of the forearm, 2 cun above the wrist crease, between the flexor carpi radialis tendon and brachioradialis tendon	Insert needle in 0.2 cun	Without needle manipulation	Deep ache or dull ache, without spreading
	Sham GV26	On the face, by the sides of GV26 1cun, above the corner of the mouth	Insert needle toward the nasal septum in 0.2 cun		
	Sham SP6	In crus inside, 3 cun above the tip of the medial malleolus, at the anterior border of the tibia, about 1.25 cun in front of SP6	Insert needle along with the medial border of the tibia in 0.2 cun		

© 2024 Li B et al. *JAMA Network Open.*

	Sham HT1	Extend the upper arm, 1 cun below the apex of the armpit, about 1 cun forward, avoiding the armpit hair, over the abdomen of the biceps	Insert needle in 0.2 cun		
	Sham LU5	Bend the elbow 120°, at the midpoint of the line between LU5 and LI11	Insert needle in 0.2 cun		
	Sham BL40	On the belly of peroneus longus, at the inferolateral of BL40. Inferior to BL40 1cun and lateral to 1cun	Insert needle in 0.2 cun		
	Sham CV23	At the rear of CV23, and by the sides of the median line 1cun	Insert needle in 0.2 cun		
	Sham beside CV23	At the rear of beside CV23, and by the sides of the median line 2cun	Insert needle in 0.2 cun		

[a]cun: 1 cun (≈25 mm) is defined as the width of the interphalangeal joint of the patient’s thumb.

© 2024 Li B et al. *JAMA Network Open.*

eTable 3. Acupuncture Intervention Details

Item	Manual acupuncture group	Sham acupuncture group
Intervention time	6 week	
Observation time	Within 6 months of onset time	
Needle retention time	30min	
Acupuncture frequency	5 times/week	
Acupuncturist	Senior professional doctors with profound background knowledge and rich experience accumulated for many years	
Materials	Disposable sterile acupuncture needles (0.25 mm × 40 mm, 0.25 mm × 75 mm; Hwato brand; Suzhou Medical Supplies Factory Co. LTD, Suzhou, China)	

© 2024 Li B et al. *JAMA Network Open.*

eTable 4. Results: Between-Group Comparison of Compliance of Participants

Compliance of participants	Manual acupuncture (n=115)	Sham acupuncture (n=116)	95% CI of Difference	*P*[a]
Compliance ＞ 80%, No. (%)	111（96.5）	114（98.3）	-	-
Completed treatment session, mean (SD)	98.20 (9.80)	99.22 (6.08)	-1.02 (-3.13 to 1.09)	0.342

[a]P value based on t test.

© 2024 Li B et al. *JAMA Network Open.*

eTable 5. Results: Treatment-by-Visit Interaction Test for the Primary Outcome[a]

	FAS		
	F	*P*	η^2
AQ			
Group effect	5.49	0.020	0.023
Visit effect	141.55	<0.001	0.803
Group*time effect	13.18	<0.001	0.226
CFCP			
Group effect	6.49	0.012	0.028
Visit effect	107.34	<0.001	0.705
Group*time effect	8.07	<0.001	0.152

[a] crude model: adjusted for none.

© 2024 Li B et al. *JAMA Network Open.*

eTable 6. Results: Adverse Event Analysis

Adverse event	Manual acupuncture (n=115)	Sham acupuncture (n=116)	*P*[a]
Participants with no adverse event, No. (%)	112 (97.4)	113 (97.4)	1.000
Participants with adverse event, No. (%)	3 (2.6)	3 (2.6)	

[a]P value based on Fisher exact test.

© 2024 Li B et al. *JAMA Network Open.*

eTable 7. Results: Adverse Event Descriptions

Clinical Sites	Randomization number	Adverse event description
First Teaching Hospital of Tianjin University of Traditional Chinese Medicine	1050	aural vertigo
Qilu Hospital of Shandong University	3003	pain after acupuncture
Qilu Hospital of Shandong University	3004	subcutaneous hematoma at CV23
Qilu Hospital of Shandong University	3005	pain after acupuncture
Qilu Hospital of Shandong University	3030	pain after acupuncture
Qilu Hospital of Shandong University	3031	subcutaneous hematoma at SP6

© 2024 Li B et al. *JAMA Network Open.*

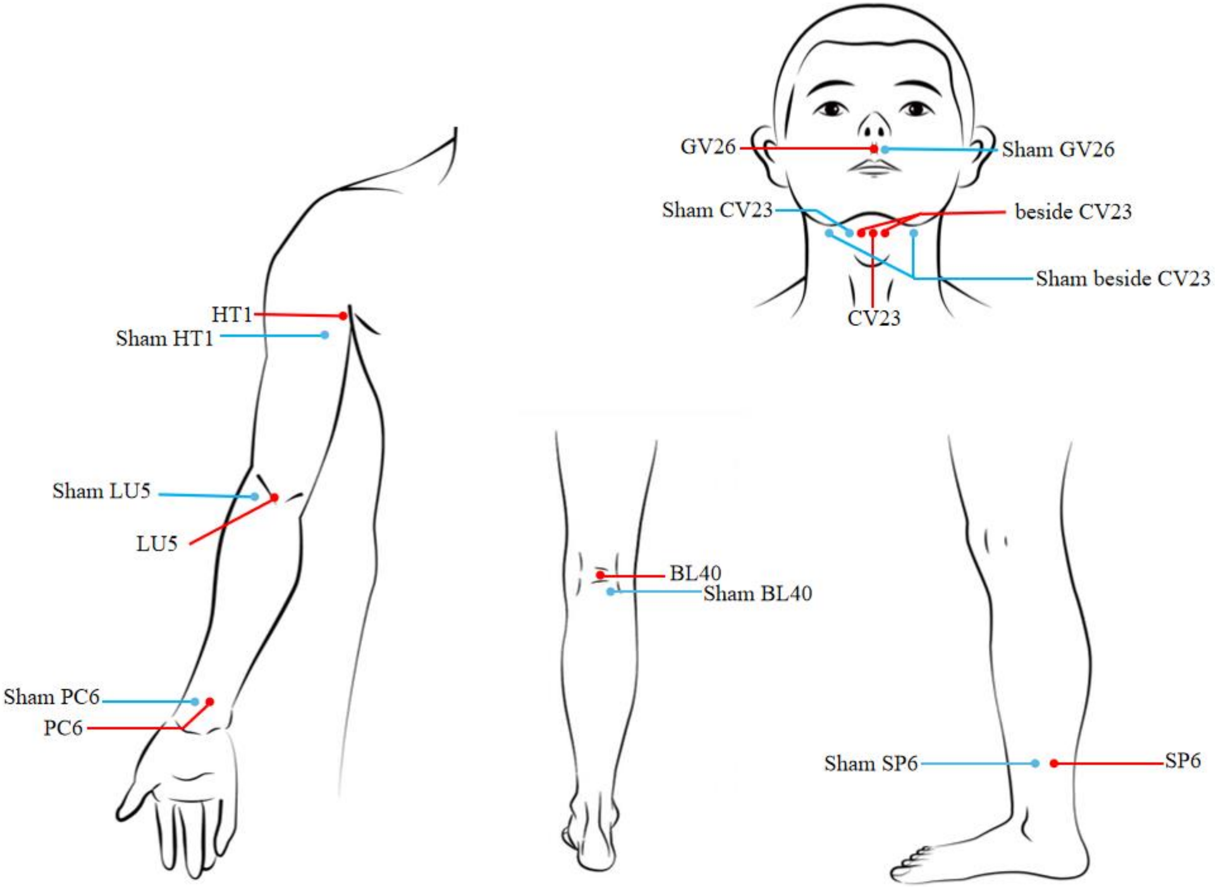

eFigure 1. Acupoint and Sham Acupoint Locations

© 2024 Li B et al. *JAMA Network Open.*

	STUDY PERIOD					
	Baseline	Intervention phase			Follow-up	
TIMEPOINT	t_0	t_{2w}	t_{4w}	t_{6w}	t_{12w}	End of 6 months after the onset time
ENROLMENT:						
Eligibility screen	X					
Basic characteristic variables	X					
Informed consent	X					
Allocation	X					
INTERVENTIONS:						
Western Aphasia Battery	X	X	X	X	X	X
Boston Diagnostic Aphasia Examination	X	X	X	X	X	X
Chinese Functional Communication Profile	X	X	X	X	X	X
National Institutes of Health Stroke Scale	X	X	X	X	X	X
Stroke and Aphasia Quality of Life Scale-39	X	X	X	X	X	X
Stroke-Specific Quality of Life Scale	X	X	X	X	X	X
Health Scale of Traditional Chinese Medicine	X	X	X	X	X	X
Combined diseases and drugs		X	X	X	X	X
Adverse event		X	X	X		
Compliance		X	X	X		

eFigure 2. Schedule of Enrollment, Interventions, and Assessments

© 2024 Li B et al. *JAMA Network Open.*

Outcome	Group	Day 15-30	Day 31-90	SMD (95%CI)	P for interaction
AQ at week 6					0.413
	Manual acupuncture	28.22 (14.99)	31.72 (12.36)	-0.25 (-0.59-0.08)	
	Sham acupuncture	16.83 (10.61)	20.61 (20.61)	-0.19 (-0.63-0.05)	
CFCP at week 6					0.681
	Manual acupuncture	72.56 (42.43)	72.87 (35.10)	-0.01 (-0.01-0.33)	
	Sham acupuncture	39.25 (35.15)	48.41 (43.87)	-0.23 (-0.56-0.11)	

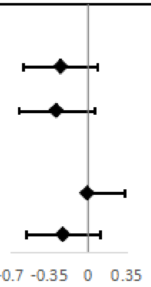

eFigure 3. Results: Subgroup Analysis. Abbreviations: AQ, aphasia quotient; CFCP, Chinese Functional Communication Profile; SMD: standardized mean difference.

© 2024 Li B et al. *JAMA Network Open.*
