# Supplementary material for: Effect of Acupuncture vs Sham Acupuncture on Patients With Poststroke Motor Aphasia: A Randomized Clinical Trial
Source: JAMA Netw Open. 2024 Jan 22;7(1):e2352580. doi: 10.1001/jamanetworkopen.2023.52580 (PMC10804271; doi:10.1001/jamanetworkopen.2023.52580)
Supplement: Supplement 3. — Data Sharing Statement [file jamanetwopen-e2352580-s003.pdf]

Data Sharing Statement

Li. Effect of Acupuncture vs Sham Acupuncture on Patients With Poststroke Motor Aphasia. *JAMA Netw Open*. Published January 22, 2024. doi:10.1001/jamanetworkopen.2023.52580

Data
Data available: Yes
Data types: Deidentified participant data, Data dictionary
How to access data: It will be made available to others upon request to the corresponding authors (profmengzhihong@163.com), only for research, and non-commercial purposes to individuals affiliated with academic or public health institutions.
When available: With publication

Supporting Documents
Document types: Statistical/analytic code, Informed consent form
How to access documents: It will be made available to others upon request to the corresponding authors (profmengzhihong@163.com), only for research, and non-commercial purposes to individuals affiliated with academic or public health institutions.
When available: With publication

Additional Information
Who can access the data: researchers whose proposed use of the data has been approved
Types of analyses: only for research
Mechanisms of data availability: with investigator support
